# Supplementary material for: Microbial cross-feeding stabilized by segregation of a dependent mutant from its independent ancestor
Source: ISME J. 2025 Jun 26;19(1):wraf131. doi: 10.1093/ismejo/wraf131 (PMC12279053; doi:10.1093/ismejo/wraf131)
Supplement: 2025_06_24_BQ_Supp_rev13_fields_wraf131 [file 2025_06_24_bq_supp_rev13_fields_wraf131.pdf]

Supplemental material for:

Microbial cross-feeding stabilized
by segregation of a dependent mutant from its independent ancestor

Short title: Rival segregation stabilizes cross-feeding

Olivia F. Schakel[1], Ryan K. Fritts[1a], Anthony J. Zmuda[1b],
Sima Setayeshgar[2]*, and James B. McKinlay[1]*
[1]Department of Biology, Indiana University, Bloomington, IN, USA
[2]Department of Physics, Indiana University, Bloomington, IN, USA

*Corresponding authors:
Setayeshgar: 727 E 3rd Street, Bloomington, IN 47405, USA; Email: simas@iu.edu
McKinlay: 1001 E 3rd Street, Bloomington, IN 47405, USA; Email: jmckinla@iu.edu

Current address:
[a] Department of Molecular, Cellular and Developmental Biology and Cooperative Institute for Research in Environmental Sciences, University of Colorado Boulder, Boulder, CO, 80309, USA
[b] Department of Plant and Microbial Biology, University of Minnesota, Twin Cities, Saint Paul, MN, 55108, USA

Mathematical model of cross-feeding.
To investigate the enrichment of a loss-of-function (LOF) mutant via mutualistic cross-feeding in bacterial cocultures, we constructed a parsimonious model of the cross-feeding between a slow-growing N_2-fixing cooperator (producer), paired with fast-growing fermentative partners that can either also fix N_2 (ancestor), or that lost N_2-fixation (LOF mutant) thereby requiring the product, ammonium (NH_4^+). The mathematical model, given by Eqs. 1-12, is a system of coupled, nonlinear reaction-diffusion equations, for three bacterial strains and nutrient levels given by organic acids, N_2, NH_4^+, and glucose, based on earlier work experimentally quantifying a mutualism between N_2-utilizing and NH_4^+-excreting *Rhodopseudomonas palustris* and NH_4^+-requiring *Escherichia coli* [1]. A minimal modification of this model to address non-privatized access to NH_4^+ production is given by Eqs. 14-18. Our experimental platform utilizes *R. palustris* as the NH_4^+-producer, and *Vibrio natriegens* as both the LOF mutant and the ancestor of the LOF mutant subpopulation, where the ancestor and can fix N_2 whereas the LOF mutant (*ΔnifA*) cannot fix N_2 and requires NH_4^+.

We numerically simulated the equations in two dimensions subject to no-flux boundary conditions using Mathematica's NDSolve [r] function employing a stiff solver (Wolfram Research, Inc., Mathematica, Version 13.3, Champaign, IL (2023)). Parameter values and diffusion constants are in Table S4. Rate constant dimensions are h^{-1}. Cell densities, c_i, are in number of cells per ml, and the numerical solution corresponds to time-dependent concentrations in a system size of $(L_x = 2 \text{ cm}) \times (L_y = 2 \text{ cm}) \times (1 \text{ cm})$. The role of spatial localization of cell densities in the $x - y$ plane is investigated, assuming the concentrations in the z −direction are uniform.

Bacterial growth in all models is given by the following Monod functions [2]:

LOF mutant growth rate (h^{-1}) (1)

$$\mu^b(G,A) = \mu^b_{max}\frac{G}{G+K^b_G}\frac{A}{A+K^b_A},$$

Ancestor growth rate on NH_4^+ (h^{-1}) (2)

$$\mu^a_A(G,A) = \mu^a_{max,A}\frac{G}{G+K^a_G}\frac{A}{A+K^a_A},$$

Ancestor growth rate on N_2 (h^{-1}) (3)

$$\mu^a_N(G,N,A) = \mu^a_{max,N}\frac{G}{G+K^a_G}\frac{N}{N+K^a_N}\left(1-\frac{A}{A+K^a_A}\right),$$

Producer growth rate on NH_4^+ (h^{-1}) (4)

$$\mu^p_A(C,A) = \mu^p_{max,A}\frac{C}{C+K^p_C}\frac{A}{A+K^p_A},$$

Producer growth rate on N_2 (h^{-1}) (5)

$$\mu^p_N(N,C,A) = \mu^p_{max,N}\frac{C}{C+K^p_C}\frac{N}{N+K^p_N}\left(1-\frac{A}{A+K^p_A}\right).$$

Additionally, in the numerical implementation, the growth terms are multiplied by a Heaviside function of cell densities, imposing a cut-off of 1 cell ml^{-1}. Related to this cut-off, in the stochastic Fisher-Kolmogorov-Petrovsky equation, which represents a broad class of models that yield nonlinear traveling waves, it has been shown that fluctuations in the wave tip are important in determining the resulting wave speed [3, 4]. Extensions of our deterministic model aim to explore stochastic effects, expected to be important in regions where cell numbers are low.

The partially privatized model uses the following equations to describe changes in population and metabolite levels:

Organic acid concentration (mM): (6)

$$\frac{\partial C}{\partial t} = D_C \nabla^2 C + F_{C,A}(\mu^b(G,A)c_b + \mu_A^a(G,A)c_a) + F_{C,N}\,\mu_N^a\,(G,N,A)c_a - (\mu_A^p(C,A)/Y_{C,A} + \mu_N^p(N,C,A)/Y_{C,N})c_p$$

Glucose concentration (mM): (7)

$$\frac{\partial G}{\partial t} = D_G \nabla^2 G - \mu_A^b(G,A)c_b/Y_{G,A} - (\mu_A^a(G,A)/Y_{G,A} + \mu_N^a(G,N,A)/Y_{G,N})c_a$$

N_2 concentration (mM): (8)

$$\frac{\partial N}{\partial t} = D_N \nabla^2 N - \mu_N^a(G,N,A)c_a/Y_N^a - \mu_N^p(N,C,A)c_p/Y_N^p$$

NH_4^+ concentration (mM): (9)

$$\frac{\partial A}{\partial t} = D_A \nabla^2 A + F_A^p \mu_N^p(N,C,A)c_P + F_A^a \mu_A^a(G,N,A)c_a - c_p \mu_A^p(C,A)/Y_A^p - c_a \mu_A^a(G,A)/Y_A^a - c_b \mu^b(G,A)/Y_A^b$$

LOF mutant cell density (cells ml^{-1}) (10)

$$\frac{\partial c_b}{\partial t} = D_b \nabla^2 c_b + \mu^b(G,A)c_b$$

Ancestor cell density (cells ml^{-1}) (11)

$$\frac{\partial c_a}{\partial t} = D_a \nabla^2 c_a + (\mu_A^a(G,A) + \mu_N^a(G,N,A))c_a$$

Producer cell density (cells ml^{-1}) (12)

$$\frac{\partial c_p}{\partial t} = D_p \nabla^2 c_p + (\mu_A^p(C,A) + \mu_N^p(N,C,A))c_p$$

The non-privatized model uses the following equations to describe changes in population and metabolite levels.

We address non-privatized access to NH_4^+ produced by the producer and the ancestor, in this case achieved via a hypothetical extracellular NH_4^+-producing enzyme, as a minimal modification to the partially privatized model equations. As part of minimizing the number of modifications, entities for N_2 and the extracellular NH_4^+-producing enzyme are not explicitly shown, and instead we assume that (i) N_2 is available at a constant saturating concentration, thus allowing Eq. 8 to be omitted, (ii) the concentration of NH_4^+-producing enzymes is proportional to populations of N_2-fixing organisms, and (iii) the rate of NH_4^+-production by the enzyme is constant and functions independently of population growth rates. Eqs. 6-12 are thus replaced with the following:

Organic acid concentration (mM): (13)

$$\frac{\partial C}{\partial t} = D_C \nabla^2 C + F_{C,A}\left(\mu^b(G,A)c_b + \mu_A^a(G,A)c_a\right) - \mu_A^p(C,A)/Y_{C,A}$$

Glucose concentration (mM): (14)

$$\frac{\partial G}{\partial t} = D_G \nabla^2 G - \mu_A^b(G,A)c_b/Y_{G,A} - \mu_A^a(G,A)c_a/Y_{G,A}$$

NH_4^+ concentration (mM): (15)

$$\frac{\partial A}{\partial t} = D_A \nabla^2 A - c_p \mu_A^p(C,A)/Y_A^p - c_a \mu_A^a(G,A)/Y_A^a - c_b \mu^b(G,A)/Y_A^b + \widetilde{F_A^p} c_P + \widetilde{F_A^a} c_a$$

LOF mutant cell density (cells ml^{-1}) (16)

$$\frac{\partial c_b}{\partial t} = D_b \nabla^2 c_b + \mu^b(G,A)c_b$$

Ancestor cell density (cells ml^{-1}) (17)

$$\frac{\partial c_a}{\partial t} = D_a \nabla^2 C_a + \mu_A^a(G,A)c_a$$

Producer cell density (cells ml^{-1}) (18)

$$\frac{\partial c_p}{\partial t} = D_p \nabla^2 c_p + \mu_A^p(C,A)c_p$$

Initial conditions with localized cell distributions.

For spatially localized initial conditions, we use Gaussian distributions with given mean and standard deviation to specify the spatial localization of cell densities:

$$c_i(x,y) = c_{0_i} e^{-\frac{(x-x_i)^2+(y-y_i)^2}{2\sigma_i^2}}. \quad (19)$$

The normalization c_{0_i} is determined to achieve a specified total initial cell count $\mathcal{N}_i(0)$ for species i for a given system size, where initial total cell numbers in simulations are chosen to be

consistent with those in the experimental chamber. Populations can be weakly or sharply localized (large or small σ_i, respectively), and the extent of co-localization is additionally determined by the mean positions, (x_i, y_i). To compare invasion from rare plots directly, where the initial frequency and the change in frequencies (horizontal and vertical axes) are specified in terms of the LOF mutant and ancestor cell numbers, the total producer population must be the same. Therefore, to compare with experimental invasion from rare measurements, in simulations we use initial producer cell numbers that are the same as those in experiments for each initial frequency.

Initial conditions with random cell distributions.

To generate random initial cell density distributions, we start with pink noise in two dimensions, which has a power spectrum of $1/k^{\alpha}$ (α = 2 in two dimensions), where $k = 2\pi/\lambda$ is the wavenumber, and λ is the wavelength. Pink noise is ubiquitous in nature and describes the statistical fluctuations of diverse physical and biological systems. The statistics of the random distribution used to generate the initial cell densities is not relevant for this analysis; rather, the focus is to address in a systematic way how successively coarsening the initial random distribution of the ancestral strain, thereby going from spatially "well-mixed" to "localized" cell densities, impacts emergence of the LOF mutant. Specifically, the pink noise random distribution is low pass filtered, attenuating wave numbers above a specified cut-off (or equivalently, attenuating the short wavelength components of the pink noise distribution). The numerical values are first clipped at zero (to achieve cell densities ≥ 0), smoothed with a Gaussian filter (to avoid sharp cell density gradients as initial conditions to the numerical simulations) and normalized to give desired total cell counts in the spatial domain. In this way, we systematically generated initial spatial density distributions displaying less point-to-point spatial variation by decreasing the filter or "fine-graining" parameter, p. The spatial coarsening that results from low pass filtering the ancestral distribution gives rise to larger regions of space where the LOF mutant is not proximal to the ancestor.

Fig. S7 shows representative initial cell densities for the LOF mutant and producer strains (Fig. S7A top row; filter parameter, $p = 0.8$) and initial cell density for the ancestor using different values of the filter parameter, demonstrating the transition from greater spatial variability (for larger filter parameter values) to lesser spatial variability (for smaller filter parameter values) (Fig. S7A, bottom row). When the LOF mutant, producer, and ancestor initial cell densities have greater spatial variability (e.g., $p = 0.8$) the populations are well-mixed and the change in LOF mutant frequency is negative (Fig. 4E, Fig. S7C), consistent with spatially uniform initial cell densities. When the ancestor is initially more localized, the LOF mutant is enriched (Fig. 4D, E; Fig. S7B).

Diffusion in agarose media.

Previous experimental work addressed diffusion of various solutes and bacterial cells in biofilms and porous media, relevant as the natural habitat for the cross-feeding dynamics considered here [5-9]. The Stokes-Einstein relation for the diffusion constant of a sphere of radius R in a low Reynolds number fluid, is given by:

$$\mathrm{D} = \frac{k_B T}{6\pi\eta \mathrm{R}}, \qquad (20)$$

where k_B is the Boltzmann constant, T is temperature and η is the fluid viscosity. While this description well represents diffusion of solutes and nonmotile cells in bulk fluid, the diffusion constant of motile cells is larger and not captured by this result [5, 6]. As a benchmark, for a sphere of radius $R \sim 1$ µm, $T = 25^{o}$C and $\eta = 0.001$ Pa · sec (water), $D \sim 2.2 \times 10^{-9}$ cm^2 s^{-1}. For rod shaped cells modeled as an ellipsoid, $R \longrightarrow R/log(2R/b)$, where R and b are the semimajor and semiminor axes, respectively [7]. For a rod-shaped cell that is 2 µm in length and 1 µm in diameter, this introduces a factor of ~ 1.4 increase in D. At 30°C, the viscosity of water is given by $\eta = 0.0008$ Pa · sec, so for diffusion of nonmotile cells in bulk fluid at the coculture temperature, we find $D \sim 3.8 \times 10^{-9}$ cm^2 s^{-1}. In our simulations, for all strains, we use $D_{cell} \sim 5 \times 10^{-9}$ cm^2 s^{-1} = 1.8×10^{-5} cm^2 h^{-1}. For the diffusion constant of small metabolites in water, ranging from 10^{-10} to 10^{-9} cm^2 s^{-1}, we use 8×10^{-10} cm^2 s^{-1} ~ 0.03 cm^2 h^{-1}.

Within a biofilm matrix, which represents a tortuous medium, diffusion is slower. Previous work measured the reduced effective diffusion constant of selected microbial solutes in biofilms [8], demonstrating a decrease by a factor of 0.2 to 0.8. In experimental studies of the rheological properties of agarose fluid gels, it has been shown that in the low agarose concentration regime, the increase in viscosity with agarose concentration is approximately linear [9]. For a 0.5% agarose solution, the viscosity at 30°C can be extracted from these measurements to be approximately 0.004 Pa · sec, which is ~5 times greater than the viscosity of water, leading to diffusion constants that correspondingly smaller by this factor [9]. These experimental results are consistent with measurements of diffusion constants in biofilms. For our numerical simulations in agarose, accounting for the increase in viscosity, we use diffusion constants that are 3-times smaller than in bulk fluid (Table S4).

Connection with Fisher-KPP equation.

The Fisher-KPP equation, due separately to Fisher [10] and Kolmogorov, Petrovsky, and Piscounov [11] is a nonlinear reaction-diffusion equation which in one dimension (Fisher's equation) can be written as

$$c_t = Dc_{xx} + \alpha c\left(1 - \frac{c}{K}\right). \tag{21}$$

The Fisher-KPP equation supports travelling wave solutions used to model spread of various invasive phenomena [12-15]. From phase plane analysis of the traveling wave solution, $c(x,t) = c(z), z = x - vt,$ it can be shown that for any $v > 0$, there exists a unique right traveling wave connecting the state $(c = K, c_x = 0)$ for $x \rightarrow -\infty$ to the state $(c = 0, c_x = 0)$ for $x \rightarrow \infty$. For $v \geq v_c$, where the critical wave speed is $v_c = 2\sqrt{\alpha D}$, the wave is nonoscillatory and a monotonically decreasing function of x. From Figure S5, which demonstrates spread of the ancestor in monoculture inoculated at one side of the chamber to the opposite end (in 0.15% agarose), we approximate a wave speed of $v \sim 2$ cm per 150 h or 0.013 cm h^{-1}. For a monotonically decreasing travelling front, requiring $v \geq v_c$ leads to $\alpha \leq v/4D$. Using our values for diffusion of cells in agarose, $D{\sim}6 \times 10^{-6}$ cm^2 h^{-1}, and $v \sim 0.013$ cm h^{-1}, we find that with a growth rate for the ancestor (*V. natriegens* grown anaerobically in minimal media with glucose) given by $\alpha \leq 0.35$ h^{-1}, this condition is readily satisfied.

Effective Growth Rate.

When cell densities are spatially varying, even if metabolite concentrations are initially uniform, they will acquire spatial structure over time. Therefore, the growth rate for each strain, which is a function of the local concentrations of metabolites at each time, will vary spatially and temporally. To assess the overall or effective growth rate of each strain at each time within the experimental system, we introduce a spatially averaged growth rate, weighted by the cell densities. For the LOF mutant and ancestor, they are given by

$$\mu^{b}_{\text{effective}}(t) = \frac{\int_{\mathcal{V}} \mu^{b}\big(G(\vec{r},t),A(\vec{r},t)\big)\, c_b(\vec{r},t)\, d^3r}{\int_{\mathcal{V}} c_b(\vec{r},t)\, d^3r}, \tag{22}$$

and

$$\mu^{a}_{\text{effective}}(t) = \frac{\int_{\mathcal{V}} \mu^{a}\big(G(\vec{r},t),A(\vec{r},t),N(\vec{r},t)\big)\, c_a(\vec{r},t)\, d^3r}{\int_{\mathcal{V}} c_a(\vec{r},t)\, d^3r}, \tag{23}$$

where the integration is over the total spatial domain $\mathcal{V}$ (in our case, the closed experimental chamber); $\mu^{b}\big(G(\vec{r},t),A(\vec{r},t)\big)$ is the LOF mutant growth rate, and $\mu^{a}\big(G(\vec{r},t),A(\vec{r},t),N(\vec{r},t)\big)$ given by

$$\mu^{a}\big(G(\vec{r},t),A(\vec{r},t),N(\vec{r},t)\big) = \mu^{a}_{A}\big(G(\vec{r},t),A(\vec{r},t)\big) + \mu^{a}_{N}\big(G(\vec{r},t),A(\vec{r},t),N(\vec{r},t)\big), \tag{24}$$

is the total ancestor growth rate (as the sum of growth rates on NH_4^+ and N_2), at each point in space at each time.

As metabolites are produced, consumed, and diffuse, the effective growth rates capture the following relevant features of growth and expansion in the coculture (visualized in Fig. S6):

i. Even if the calculated growth rate $\mu^{i}(\vec{r},t)$ (where $i = a, b$ refers to the ancestor or LOF mutant) is high in a given spatial region but the ancestor/LOF mutant is not present at that location at that time (that is, $c_i(\vec{r},t) \approx 0$), the effective growth rate $\mu^{i}_{\text{effective}}(t)$ will not be high.
ii. Spatial separation of the LOF mutant and ancestor allows the LOF mutant to grow in regions where it is not directly competing with the ancestor: even if $\mu^{a} > \mu^{b}$ in such a region, the ancestor does not outcompete the LOF mutant because it is not present to take advantage of this higher growth rate.
iii. Non-motile cells do not diffuse quickly through agarose, avoiding competition, whereas NH_4^+ diffuses quickly enough from the producer to support appreciable LOF mutant effective growth in regions where the ancestor is not present.
iv. In the absence of motility, slow diffusion of cells implies that by the time the ancestor reaches spatial pockets where the LOF mutant has experienced growth, glucose may be completely depleted, halting the ancestor's further growth and expansion.

To illustrate these points, in Fig. 6B and C, starting with a small initial frequency for the LOF mutant ($f_0 = 0.061$), we plot cross-sections of the ancestor and LOF mutant cell density profiles ($c_a(\vec{r},t)$ and $c_b(\vec{r},t)$), and growth rates ($\mu^{a}(\vec{r},t)$ and $\mu^{b}(\vec{r},t)$), at $y = 1$ cm at $t = 10$ h. We

note that on the right side of the chamber where the ancestor is localized, its growth rate is approximately zero given that glucose is depleted. On the left side of the chamber, the ancestor growth rate is high (given the availability of glucose, NH_4^+ and N_2); however, the ancestor is not present in this region to take advantage of the high growth rate. Therefore, the effective ancestor growth rate at 10 h is small. On the other hand, the peak of the LOF mutant cell density is located in a region where its growth rate is high; consequently, the effective growth rate is comparable and high.

LOF change in frequency.

The metric used to quantify emergence of the loss of function mutant in invasion from rare assays is the change in frequency defined as

$$\Delta f(t) = f(t) - f(0), \tag{25}$$

where $f(t)$ is the LOF mutant frequency at time t, and $f_0 = f(0)$ is the frequency at $t = 0$. $f(t)$ is given by

$$f(t) = \frac{\mathcal{N}_b(t)}{\mathcal{N}_b(t)+\mathcal{N}_a(t)}, \tag{26}$$

and $\mathcal{N}_{a,b}(t)$ is the total number of ancestor/LOF mutant cells in the chamber at time t,

$$\mathcal{N}_{a,b}(t) = \int_{\mathcal{V}} c_{a,b}(\vec{r},t)\ d^3r. \tag{27}$$

Therefore,

$$\Delta f(t) = \frac{\mathcal{N}_b(t)}{\mathcal{N}_b(t)+\mathcal{N}_a(t)} - \frac{\mathcal{N}_b(0)}{\mathcal{N}_b(0)+\mathcal{N}_a(0)}, \tag{28}$$

Note that the time rate of change of total cell numbers is:

$$\frac{d\mathcal{N}_a}{dt} = \int_{\mathcal{V}} \mu^a\big(G(\vec{r},t), A(\vec{r},t), N(\vec{r},t)\big)\ c_a(\vec{r},t)\, d^3r. \tag{29}$$

and

$$\frac{d\mathcal{N}_b}{dt} = \int_{\mathcal{V}} \mu^b\big(G(\vec{r},t), A(\vec{r},t)\big)\ c_b(\vec{r},t)\, d^3\mathrm{r}. \tag{30}$$

From the definition of the effective growth rates, given by Eq. (22)-(23), we have

$$\frac{d\mathcal{N}_{a,b}}{dt} = \mathcal{N}_{a,b}(t)\mu^{a,b}_{\text{effective}}(t), \tag{31}$$

and integrating we can find

$$\mathcal{N}_{a,b}(t) = \mathcal{N}_{a,b}(0)e^{\int_0^t \mu^{a,b}_{\text{effective}}(t')dt'}, \tag{32}$$

where the overall exponential growth of the ancestor/LOF mutant is given by the time integral of the effective growth rate

$$\int_0^t \mu_{\text{effective}}^{a,b}(t')dt'. \tag{33}$$

Therefore, the change in frequency can be written as

$$\Delta f(t) = \frac{\mathcal{N}_b(0)}{\mathcal{N}_b(0)+\mathcal{N}_a(0)\, e^{\int_0^t \left(\mu_{\text{effective}}^a(t')-\mu_{\text{effective}}^b(t')\right)dt'}} - \frac{\mathcal{N}_b(0)}{\mathcal{N}_b(0)+\mathcal{N}_a(0)}. \tag{34}$$

From Eq. (34) we can see that whether the change in frequency, $\Delta f(t)$, is positive or negative depends on the difference in the overall growth. This can be summarized as:

i. Difference in overall growth between ancestor and the LOF mutant is positive:

$$\int_0^t \left(\mu_{\text{effective}}^a(t') - \mu_{\text{effective}}^b(t')\right) dt' > 0 \Rightarrow e^{\int_0^t \left(\mu_{\text{effective}}^a(t')-\mu_{\text{effective}}^b(t')\right)dt'} > 1 \Rightarrow f(t) < f(0), \tag{35}$$

ii. Difference in overall growth between ancestor and the LOF mutant is negative:

$$\int_0^t \left(\mu_{\text{effective}}^a(t') - \mu_{\text{effective}}^b(t')\right) dt' < 0 \Rightarrow e^{\int_0^t \left(\mu_{\text{effective}}^a(t')-\mu_{\text{effective}}^b(t')\right)dt'} < 1 \Rightarrow f(t) > f(0). \tag{36}$$

Figure S9 show the effective growth rates for the LOF mutant and ancestor (top panel for each time point), their time integrals (lower panel for each time point) and the change in frequency (right panel for each time point). The vertical lines in each plot refer to the time point t at which the change in frequency is considered. The shaded area under each curves is the time integral of the effective growth rate up to that time point; this area corresponds to the overall growth at that time, and marked by the dashed line in the lower panels. These figures demonstrate the fact that initially (Fig. S9, 2 h), the overall growth of the ancestor is greater than that of the LOF mutant and the change in frequency is negative; however, at later times (Fig. S9, 12.5 h), as glucose in the vicinity of the localized ancestor cell density becomes depleted (Fig. S8), the overall growth of the LOF mutant surpasses that of the ancestor, and the change in frequency becomes positive. When the overall growths are equal, the change in frequency is zero (Fig. S9, 5.5 h).

Table S1. Strains

Strain	Designation; genotype; phenotype	Source or reference
R. palustris strains		
CGA0092	Wild-type; spontaneous Cm^R derivative of CGA001	[16, 17]
CGA4039	Δnitrogenase or ΔN_2ase; nifA*, Δ*uppE* (RPA2750), Δ*nifH*, Δ*vnfH,* Δ*anfH*; incapable of N_2 fixation	This study
CGA4005	*nifA**, Δ*hupS*, Δ*uppE*; constitutive nitrogenase expression resulting in NH_4^+ excretion, incapable of H_2 oxidation, decreased capacity for biofilm formation	[1]
CGA4067	Producer; *nifA**, Δ*hupS*, Δ*uppE*, Δ*motAB*; NH_4^+ excretion, incapable of H_2 oxidation, decreased capacity for biofilm formation, non-motile due to absence of flagellar stator	This study
V. natriegens strains		
TND1964	Wild type (WT); ATCC14048, pMMB-tfoX	[18]
NH003	WT Δ*dns*::Kan^R, pMMB-tfoX	This study
NH004	WT Δ*dns*::$Spec^R$, pMMB-tfoX	This study
OFS003	Ancestor; Δ*dns*::Kan^R, pMMB-tfoX, Δ*flgE,*; non-motile due to the absence of the flagellar hook	This study
OFS004	LOF mutant; Δ*dns*::$Spec^R$, pMMB-tfoX, Δ*flgE,* Δ*nifA*; non-motile and incapable of N_2 fixation	This study
OFS005	pMMB-tfoX, Δ*nifA::*$Spec^R$; incapable of N_2 fixation	This study

Table S2. Plasmids

Plasmid	Description	Source or reference
pJQ200SK	Suicide vector used for making deletion mutants in *R. palustris*	[19]
pJQΔ*motAB*	Suicide vector for deleting the flagellar stator genes in *R. palustris*	[20]
pJQΔ*nifH*	Suicide vector for deleting the Mo-nitrogenase gene in *R. palustris*	[21]
pJQΔ*vnfH*	Suicide vector for deleting the V-nitrogenase gene in *R. palustris*	[21]
pJQΔ*anfH*	Suicide vector for deleting the Fe-nitrogenase gene in *R. palustris*	[21]
pMMB-tfoX	Expression vector with IPTG-inducible *V. cholerae tfoX* to allow for natural transformation.	[18]

Table S3. Primers

Primer	Sequence	Description	Source or reference
OFS64	gatatcgaaatgccagagatggac	Δ*flgE* forward, 3 kb upstream	This study
OFS65	gtcgacggatccccggaattccaaagtct cctgatctcgc	Δ*flgE* MUGENT MiniFRT overlap reverse	This study
OFS66	agaagcagctccagcctacaatcgcga gccggataatc	Δ*flgE* MUGENT MiniFRT overlap forward	This study
OFS67	acggttgggttattaccgacaatc	Δ*flgE* reverse, 3 kb downstream	This study
OFS70	gacttgcagaagtattggaagtcg	Δ*flgE* sequencing forward	This study
OFS71	gtcataaagtaatcaagtgagctgc	Δ*flgE* sequencing reverse	This study
JPL007	gagataagtgacgcaagccg	Δ*nifA* reverse, 3 kb downstream	This study
JPL006	gaagcagctccagcctacacaaggctg ctcaatatgacgc	Δ*nifA* MUGENT MiniFRT overlap forward	This study
JPL005	gtcgacggatccccggaatccgccaata actgacgctc	Δ*nifA* MUGENT MiniFRT overlap reverse	This study
SB001	ggacatgattcactcctaaaccg	Δ*nifA* forward, 3 kb downstream	This study
JPL48	atgatagaagatcattctc	Δ*nifA* sequencing forward	This study
JPL49	tcagatttgtttcatctcgatatt	Δ*nifA* sequencing reverse	This study
NH022	cgaggtgaagatcattcatttcc	Δ*dns* forward, 1 kb upstream	This study
NH025	cttagtgattgggtcactcattgg	Δ*dns* reverse, 1 kb upstream	This study
NH014	ctctgcaccactaccgtc	Δ*dns* sequencing forward	This study
NH015	cgaataccgatgtcgctgc	Δ*dns* sequencing reverse	This study
Prm361	attccggggatccgtcgacctgcagttca gaagcagctccagcctaca	miniFRT cassette forward	[22]
Prm362	tgtaggctggagctgcttctgaactgcag gtcgacggatccccggaat	miniFRT cassette reverse	[22]

Table S4. Model parameter values.

Parameter	Description	Value	Units
Maximum growth rates			
$\mu^{a}_{max,A}$	ancestor on NH_4^+, measured, this study	0.348[a]	h^{-1}
$\mu^{b}_{max,A}$	LOF mutant on NH_4^+, measured, this study	0.348 $\times$ 1.1, 1.3	h^{-1}
$\mu^{p}_{max,A}$	producer on NH_4^+, [23]	0.0875	h^{-1}
$\mu^{a}_{max,N}$	ancestor on N_2, measured, this study	0.222[a]	h^{-1}
$\mu^{p}_{max,N}$	producer on N_2, measured [1]	0.0772	h^{-1}
Metabolite production levels and rates			
$F_A^{p,a}$	NH_4^+ excretion level by producer, ancestor in privatized model, estimated, this study	6.5×10^{-10}	µmol $cell^{-1}$
$\widetilde{F_A^{p,a}}$	NH_4^+ excretion rate by producer, ancestor in non-privatized model, estimated, this study	6.5×10^{-10}	µmol $cell^{-1}$ s^{-1}
F_C	organic acid production level during growth on NH_4^+ by ancestor, LOF mutant, measured, this study	1.0×10^{-6}	µmol $cell^{-1}$
$F_{C,N}^{a}$	organic acid production level during growth on N_2 by ancestor, measured, this study	1.95×10^{-6}	µmol $cell^{-1}$
Half-saturation constants			
$K_A^{a,b}$	ancestor, LOF mutant affinity for NH_4^+, assumed [24]	0.01	mM
K_A^{p}	producer affinity for NH_4^+, assumed [25]	0.1	mM
$K_G^{a,b}$	ancestor, LOF mutant affinity for glucose, assumed [26]	0.02	mM
K_C^{p}	producer affinity for organic acids, assumed	0.01	mM
$K_N^{a,p}$	ancestor, producer affinity for N_2, measured [27]	6	mM
Growth yields			
Y_N^{p}	producer from N_2, measured [27]	5×10^{8}	cell $µmol^{-1}$
Y_N^{a}	ancestor from N_2, measured	4×10^{8}	cell $µmol^{-1}$
Y_G	ancestor, LOF mutant from glucose with NH_4^+, measured	1.9×10^{6}	cell $µmol^{-1}$
$Y_{G,N}^{a}$	ancestor from glucose with N_2, measured	1×10^{6}	cell $µmol^{-1}$
$Y_A^{a,b}$	ancestor, LOF mutant from NH_4^+, measured	2×10^{8}	cell $µmol^{-1}$
Y_A^{p}	producer from NH_4^+, assumed	5×10^{8}	cell $µmol^{-1}$
$Y_{C,N}^{p}$	producer from organic acids with N_2, measured	1.3×10^{8}	cell $µmol^{-1}$
$Y_{C,A}^{p}$	producer from organic acids with NH_4^+, assumed	1.3×10^{8}	cell $µmol^{-1}$
Diffusion constants in liquid			
D_a, D_b, D_p	non-motile ancestor, LOF mutant, producer cells	1.8×10^{-5}	cm^2 h^{-1}
D_C, D_G, D_A, D_N	organic acids, glucose, NH_4^+, N_2	0.03	cm^2 h^{-1}
Diffusion constants in 0.15% agarose			
D_a, D_b, D_p	non-motile ancestor, LOF mutant, producer cells	6×10^{-6}	cm^2 h^{-1}
D_C, D_G, D_A, D_N	organic acids, glucose, NH_4^+, N_2	0.01	cm^2 h^{-1}
Default initial values			
C	organic acids	0.000001	mM
G	glucose	5	mM
A	NH_4^+	0.00005	mM
N	N_2; assumed to be fully dissolved	70	mM
c_i	cell density	varied[b]	cells ml^{-1}

[a] To avoid our interpretations being influenced by the extreme growth rates of *V. natriegens* we ran simulations with values corresponding to a slower-growing fermentative N_2-fixing bacterium, *Zymonomas mobilis* [28].

[b] Simulated initial cell densities were the same as those measured in experimental assays.

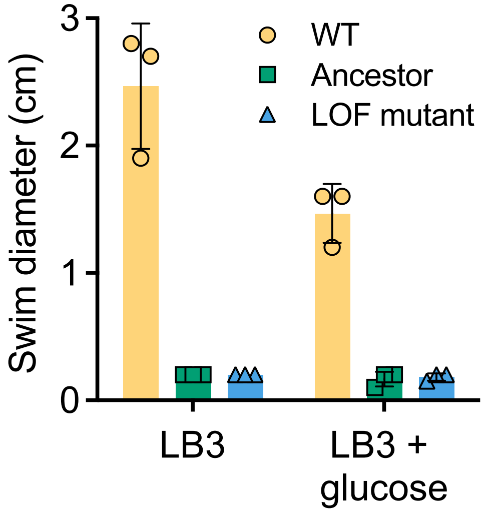

Figure S1. Deleting *V. natriegens flgE* eliminates motility; two kinds of media were used to assess whether 10 mM glucose would fully inhibit motility; bars are the mean of three biological replicates ± SD. WT, NH003.

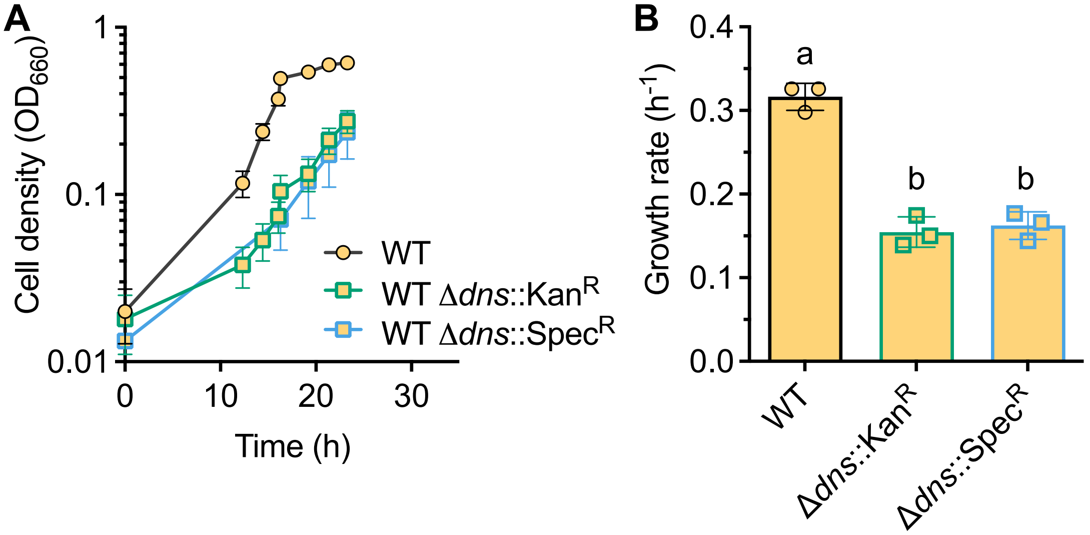

Figure S2. Δ*dns*::Kan^R and Δ*dns*::Spec^R have equal effects on growth rate; (A) *V. natriegens* monoculture growth curves when *dns* is intact or replaced with different antibiotic cassettes; (B) monoculture growth rates from (A); floating letters indicate significant differences from a one-way ANOVA with Tukey's multiple comparison post-test ($P > 0.05$); (A, B) points or bars are means ± SD, n = 3.

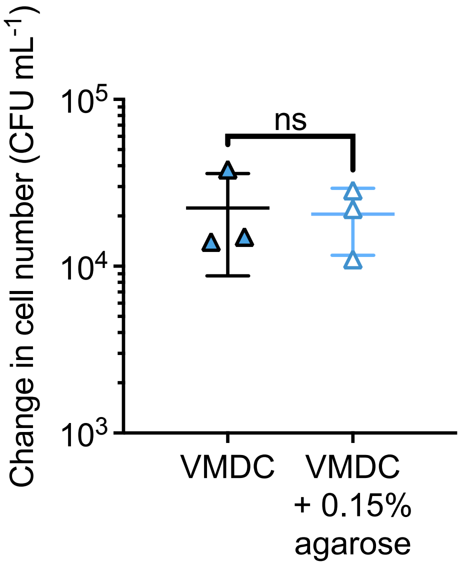

Figure S3. Washed 0.15% agarose does not contain enough nitrogen to support growth of the *V. natriegens* LOF mutant (OFS005); points are individual cultures; bar is mean ± SD, n=3; ns = non-significat based on a two-tailed unpaired t-test (p = 0.85).

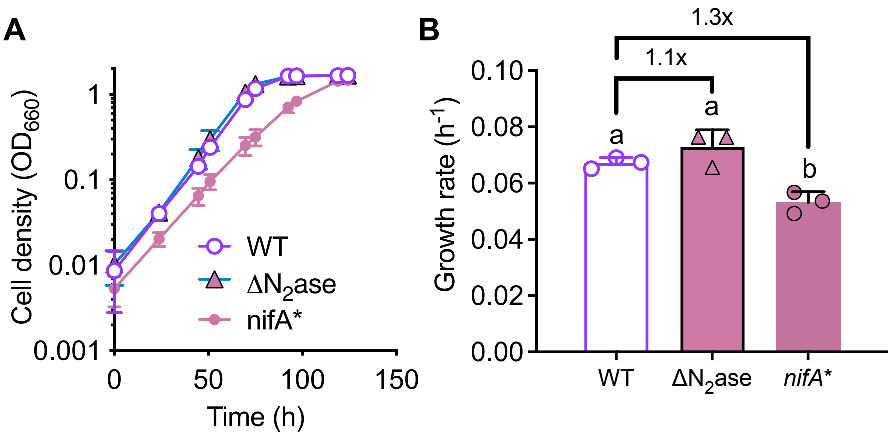

Figure S4. Estimation of growth advantage imparted by loss of nitrogenase (A) growth of *R. palustris* CGA0092 (WT), CGA4039 (Δnitrogenase; ΔN_2ase) (CGA4005 (*nifA**) in media with NH_4Cl; (B) growth rates calculated from the exponential phase in (A); the floating values indicate fold-differences in growth rate; floating letters indicate significant differences according to a one-way ANOVA with Tukey's multiple comparisons test ($P > 0.05$); a similar comparison with *V. natriegens* was not possible as we have yet to successfully generate a NifA* strain.

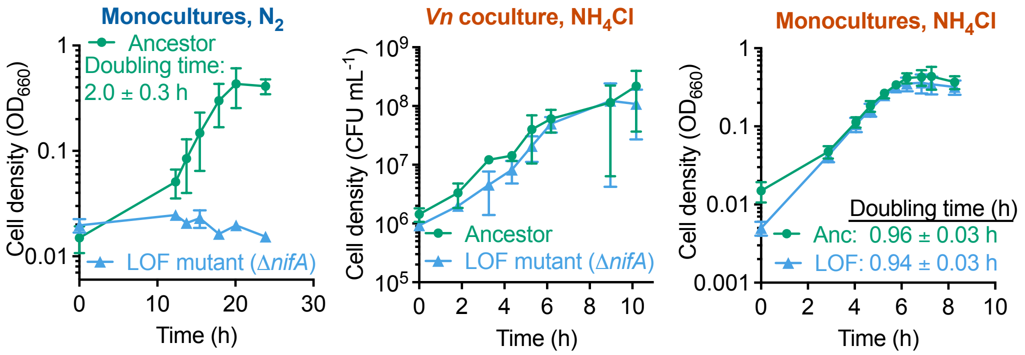

Figure S5. The *V. natriegens* Δ*nifA* LOF mutant cannot use N_2 but the ancestor (Anc) and LOF mutant growth rates are similar with NH_4Cl; (A) monoculture growth with N_2 as the nitrogen source; data points and doubling time are means ± SD, n = 3; (B) growth of the ancestor and the LOF mutant in cocultures with 10 mM NH_4Cl; data points are means ± SD n = 3; *R. palustris* was not included in these cocultures; (C) monoculture growth with 10 mM NH_4Cl; data points and doubling time are means ± SD, n = 3; p = 0.59 (two-tailed t-test).

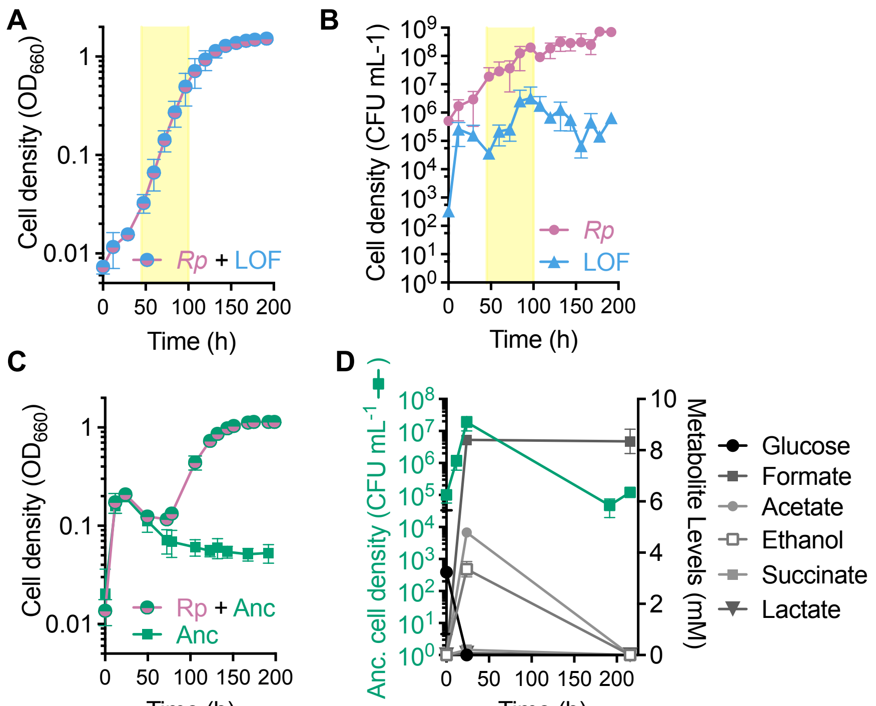

Figure S6. *R. palustris* Nx 'producer' (*Rp*) and the *V. natriegens* Δ*nifA* LOF mutant grow by reciprocal cross-feeding; (A, B) growth of cocultures pairing the producer and the LOF mutant, tracked using coculture turbidity (A) or colony forming units for each population (B); the exponential phase is highlighted in yellow; (C) comparison of growth curves between WT *V. natriegens* 'ancestor' (Anc) monocultures and cocultures pairing the ancestor with the producer; (D) glucose and fermentation product concentrations in ancestor + producer cocultures; (A-D) data points are means ± SD, n = 3.

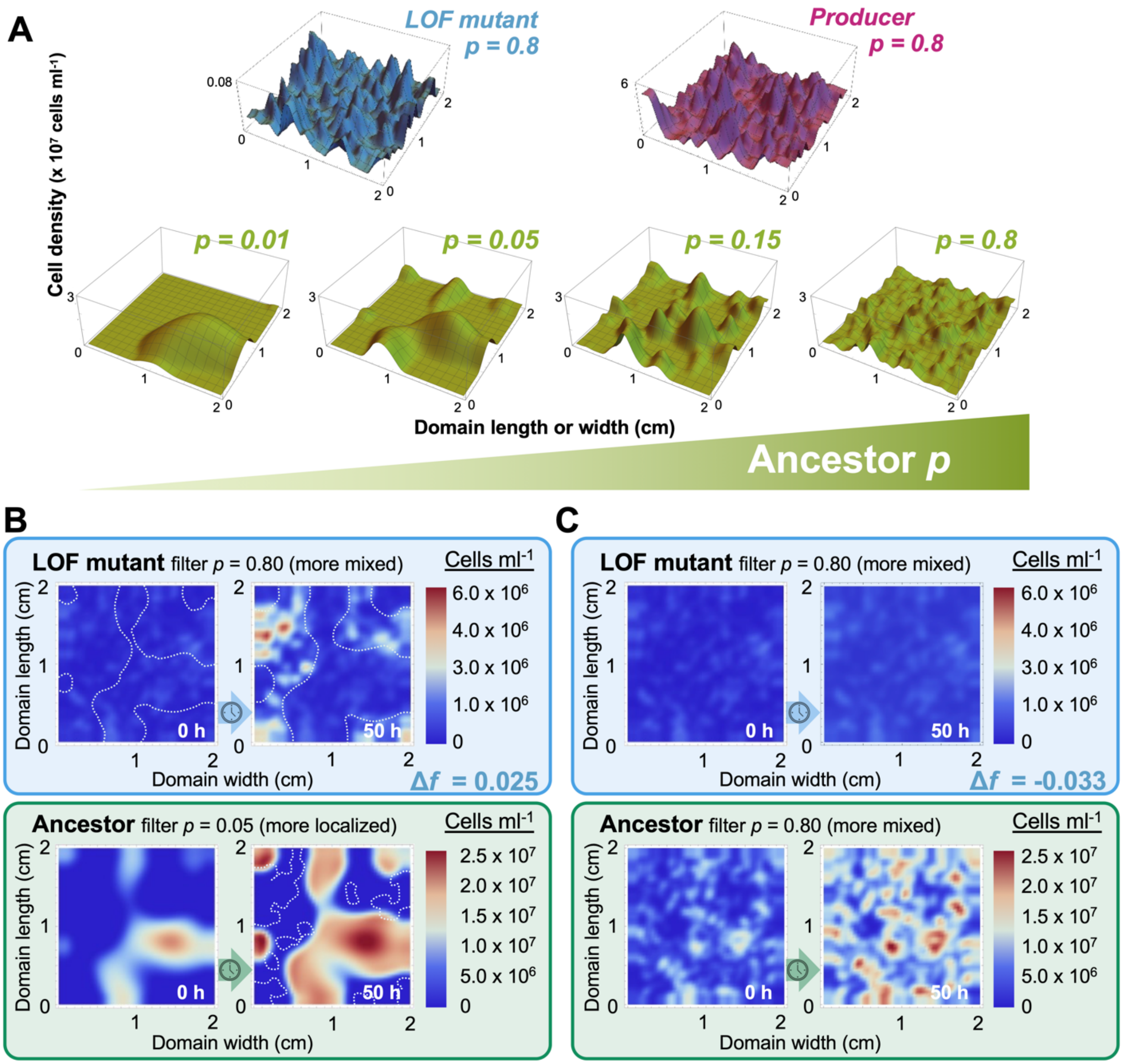

Figure S7. The degree to which initial ancestor spatial cell densities are randomized affects whether the LOF mutant is enriched; (A-C) initial LOF mutant frequency $f_0 = 0.061$; LOF mutant and producer initial cell densities were held fixed with a filter parameter $p = 0.8$; (A) upper row, an example of randomized initial LOF mutant and producer cell densities; lower row, an example of how the same ancestor initial cell density is randomly distributed using different spatial filter values, p; the ancestor spatial cell density becomes more fine-grained and well-mixed with the other population with increasing p; (B-C) examples of how a low ancestor spatial filter (B, $p = 0.05$, more localized) can lead to LOF mutant enrichment whereas a higher ancestor spatial filter (C, $p = 0.80$, more mixed), prevents LOF mutant enrichment; panel B is the same data as in Fig. 4D; dotted lines show the boundaries where the competitor population is relatively high (compare top vs bottom graphs).

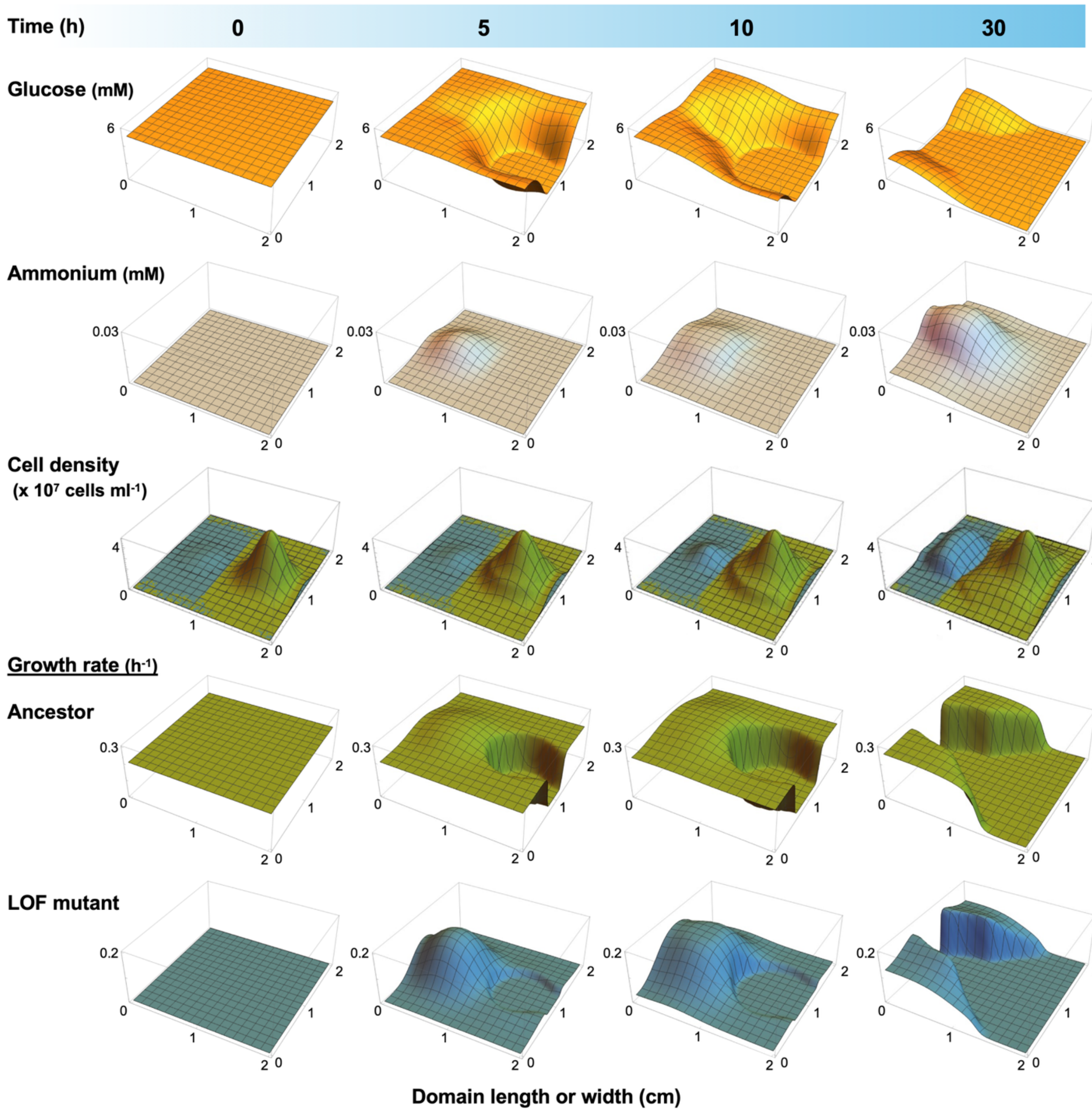

Figure S8. Snapshots from simulations where the producer and LOF mutant (blue) are colocalized away from the ancestor (green) (σ = 0.2). Glucose is quickly depleted at the ancestor inoculation site (5 h). The LOF mutant consumes enough glucose near its inoculation site to slow the advance of the ancestor into its territory (10 h). Both populations the expand to the sides where glucose remains. Note that the growth rate plots depict the maximum theoretically possible growth rates at a given location based on availability of metabolites. In some cases, the regions with a high theoretical maximum growth rate have few if any cells present to take advantage of the growth conditions.

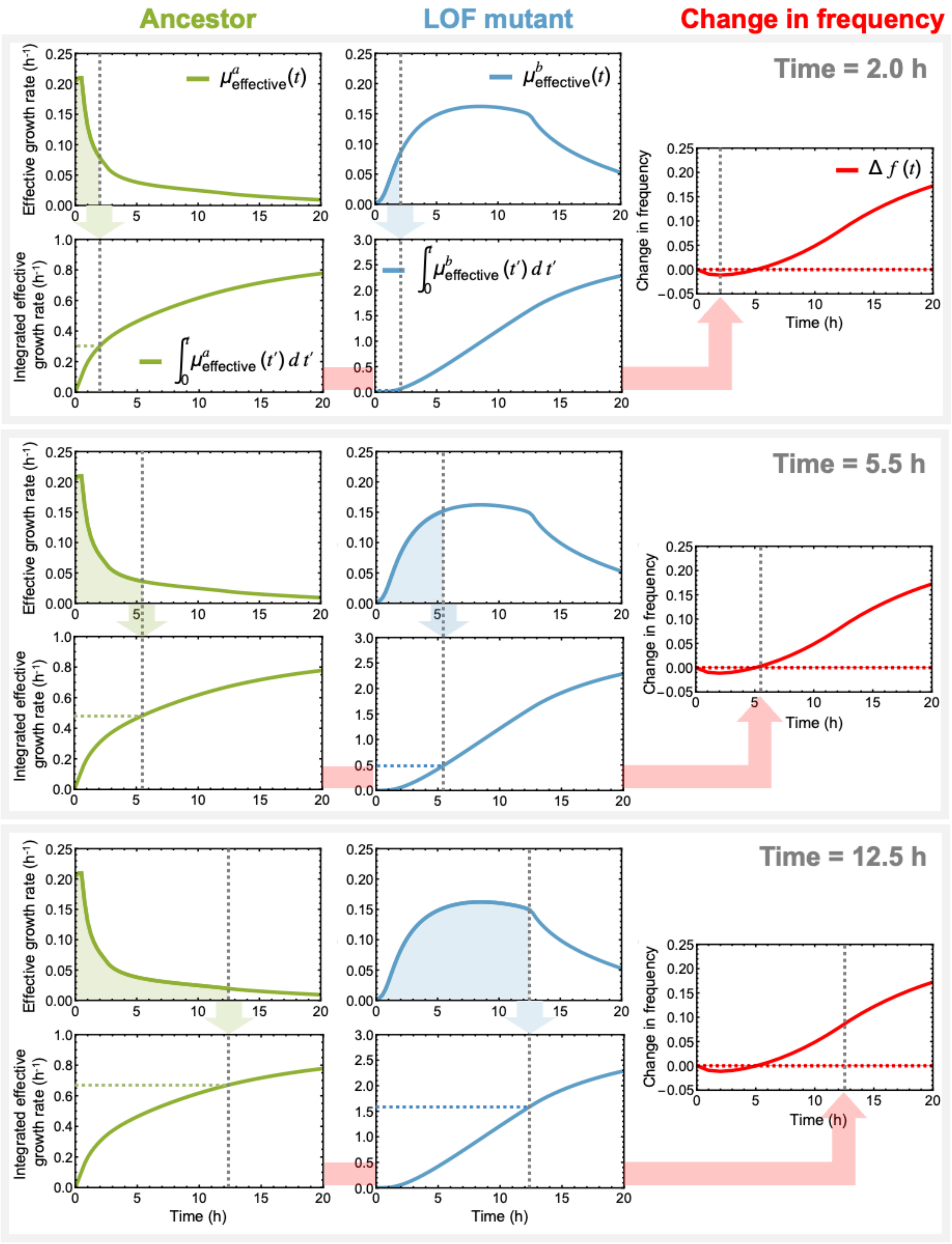

Figure S9. Change in LOF mutant frequency ($\Delta f(t)$) can be calculated by integrating the effective growth rate of each *V. natriegens* population; three time points are shown (2.0, 5.5, and 12.5 h) to illustrate how the change in frequency plot is built; shaded regions are the integrated effective growth rates up to that time point, leading to a data point in the graph below (follow the shaded green and blue arrows); the integrated effective growth rates are then used to calculate the change in frequency (follow the shaded red arrows); for more details see the description of Eqs. (22)-(36), above.

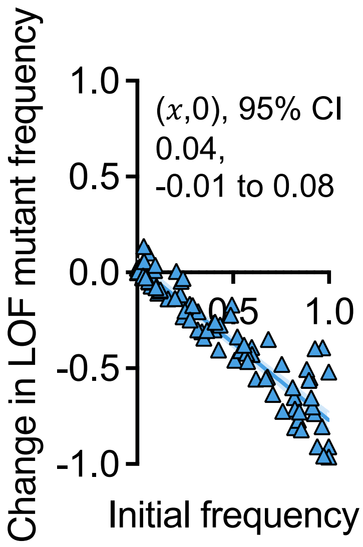

Figure S10. Compilation of all data from every IFR experiment performed in static liquid; each point is a single experimental coculture; initial frequency range = 0.1% - 99.9% using *V. natriegens* populations only; x-intercept and 95% CI error bands were determined using linear regression analysis.

References for the supplemental material.

1. LaSarre B, McCully AL, Lennon JT, McKinlay JB. Microbial mutualism dynamics governed by dose-dependent toxicity of cross-fed nutrients. *ISME J* 2017;**11**:337-48. https://doi.org/10.1038/ismej.2016.141
2. Allen RJ, Waclaw B. Bacterial growth: a statistical physicist's guide. *Rep Prog Phys* 2019;**82**:016601. https://doi.org/10.1088/1361-6633/aae546
3. Brunet E, Derrida B. Shift in the velocity of a front due to a cutoff. *Physical Review E* 1997;**56**:2597. https://doi.org/DOI: https://doi.org/10.1103/PhysRevE.56.2597
4. Dumortier F, Popović N, Kaper T. The critical wave speed for the Fisher-Kolmogorov-Petrowskii-Piscounov equation with cut-off. *Nonlinearity* 2007;**20**:855. https://doi.org/DOI 10.1088/0951-7715/20/4/004
5. Licata NA, Mohari B, Fuqua C, Setayeshgar S. Diffusion of bacterial cells in porous media. *Biophys J* 2016;**110**:247-57. https://doi.org/10.1016/j.bpj.2015.09.035
6. Bhattacharjee T, Datta SS. Bacterial hopping and trapping in porous media. *Nat Commun* 2019;**10**:2075. https://doi.org/10.1038/s41467-019-10115-1
7. Berg H. *Random walks in biology*. Princeton University Press., 1993.
8. Stewart PS. Diffusion in biofilms. *J Bacteriol* 2003;**185**:1485-91. https://doi.org/10.1128/JB.185.5.1485-1491.2003
9. Ghebremedhin M, Seiffert S, Vilgis TA. Physics of agarose fluid gels: Rheological properties and microstructure. *Curr Res Food Sci* 2021;**4**:436-48. https://doi.org/10.1016/j.crfs.2021.06.003
10. Fisher RA. The wave of advance of advantageous genes. *Ann Eugen* 1937;**4**:353-9.
11. Kolmoforov A, Petrovsky I, Piscunov N. Etude de l'equation de la diffusion avec croissance de la quantite de matiere et son application a un probleme biologique. *Moscou Universitet Bull Math* 1937;**1**
12. Colon JG, Doering CR. On travelling waves for the stochastic Fisher-Kolmogorov-Petrovsky-Piscunov equation. *J Stat Phys* 2005;**120**:421-77.

13. Houchmandzadeh B, Vallade M. Fisher waves: An individual-based stochastic model. *Phys Rev E* 2017;**96**:012414. https://doi.org/10.1103/PhysRevE.96.012414
14. Hallatschek O. The noisy edge of traveling waves. *Proc Natl Acad Sci U S A* 2011;**108**:1783-7. https://doi.org/10.1073/pnas.1013529108
15. Venegas-Ortiz J, Allen RJ, Evans MR. Speed of invasion of an expanding population by a horizontally transmitted trait. *Genetics* 2014;**196**:497-507. https://doi.org/10.1534/genetics.113.158642
16. Larimer FW, Chain P, Hauser L, Lamerdin J, Malfatti S, Do L *et al.* Complete genome sequence of the metabolically versatile photosynthetic bacterium *Rhodopseudomonas palustris*. *Nat Biotechnol* 2004;**22**:55-61. https://doi.org/10.1038/nbt923
17. Mazny BE, Sheff OF, LaSarre B, McKinlay A, McKinlay JB. Complete genome sequence of *Rhodopseudomonas palustris* CGA0092 and corrections to the *R. palustris* CGA009 genome sequence. *Microbiol Resour Announc* 2023;**12**:e0128522. https://doi.org/10.1128/mra.01285-22
18. Dalia TN, Hayes CA, Stolyar S, Marx CJ, McKinlay JB, Dalia AB. Multiplex genome editing by natural transformation (MuGENT) for synthetic biology in *Vibrio natriegens*. *ACS Synth Biol* 2017;**6**:1650-5. https://doi.org/10.1021/acssynbio.7b00116
19. Quandt J, Hynes MF. Versatile suicide vectors which allow direct selection for gene replacement in gram-negative bacteria. *Gene* 1993;**127**:15-21. https://doi.org/10.1016/0378-1119(93)90611-6
20. Fritts RK. The physiology, mechanisms, and evolution of bacterial cross-feeding and nutrient acquisition. Biology, Indiana University, 2019
21. Oda Y, Samanta SK, Rey FE, Wu L, Liu X, Yan T *et al.* Functional genomic analysis of three nitrogenase isozymes in the photosynthetic bacterium *Rhodopseudomonas palustris*. *J Bacteriol* 2005;**187**:7784-94. https://doi.org/10.1128/JB.187.22.7784-7794.2005
22. Dalia AB, Seed KD, Calderwood SB, Camilli A. A globally distributed mobile genetic element inhibits natural transformation of *Vibrio cholerae*. *Proc Natl Acad Sci U S A* 2015;**112**:10485-90. https://doi.org/10.1073/pnas.1509097112
23. Chuang YC, Haas NW, Pepin R, Behringer MG, Oda Y, LaSarre B *et al.* Bacterial adenine cross-feeding stems from a purine salvage bottleneck. *ISME J* 2024;**18**:wrae034. https://doi.org/10.1093/ismejo/wrae034
24. Khademi S, O'Connell J, 3rd, Remis J, Robles-Colmenares Y, Miercke LJ, Stroud RM. Mechanism of ammonia transport by Amt/MEP/Rh: structure of AmtB at 1.35 A. *Science* 2004;**305**:1587-94. https://doi.org/10.1126/science.1101952
25. McCully AL, LaSarre B, McKinlay JB. Recipient-biased competition for an intracellularly generated cross-fed nutrient is required for coexistence of microbial mutualists. *mBio* 2017;**8**:e01620-17. https://doi.org/10.1128/mBio.01620-17
26. Buhr A, Daniels GA, Erni B. The glucose transporter of *Escherichia coli*. Mutants with impaired translocation activity that retain phosphorylation activity. *J Biol Chem* 1992;**267**:3847-51.
27. McCully AL, LaSarre B, McKinlay JB. Growth-independent cross-feeding modifies boundaries for coexistence in a bacterial mutualism. *Environ Microbiol* 2017;**19**:3538-50. https://doi.org/10.1111/1462-2920.13847
28. Kremer TA, LaSarre B, Posto AL, McKinlay JB. N_2 gas is an effective fertilizer for bioethanol production by *Zymomonas mobilis*. *Proc Natl Acad Sci U S A* 2015;**112**:2222-6. https://doi.org/10.1073/pnas.1420663112
